# Epistasis in a Model of Molecular Signal Transduction

**DOI:** 10.1371/journal.pcbi.1001134

**Published:** 2011-05-12

**Authors:** Alain Pumir, Boris Shraiman

**Affiliations:** 1 Laboratoire de Physique, Ecole Normale Supérieure de Lyon, Lyon, France; 2 Kavli Institute for Theoretical Physics and Department of Physics, University of California, Santa Barbara, California, United States of America; Stanford University, United States of America

## Abstract

Biological functions typically involve complex interacting molecular networks, with numerous feedback and regulation loops. How the properties of the system are affected when one, or several of its parts are modified is a question of fundamental interest, with numerous implications for the way we study and understand biological processes and treat diseases. This question can be rephrased in terms of relating genotypes to phenotypes: to what extent does the effect of a genetic variation at one locus depend on genetic variation at all other loci? Systematic quantitative measurements of epistasis – the deviation from additivity in the effect of alleles at different loci – on a given quantitative trait remain a major challenge. Here, we take a complementary approach of studying theoretically the effect of varying multiple parameters in a validated model of molecular signal transduction. To connect with the genotype/phenotype mapping we interpret parameters of the model as different loci with discrete choices of these parameters as alleles, which allows us to systematically examine the dependence of the signaling output – a quantitative trait – on the set of possible allelic combinations. We show quite generally that quantitative traits behave approximately additively (weak epistasis) when alleles correspond to small changes of parameters; epistasis appears as a result of large differences between alleles. When epistasis is relatively strong, it is concentrated in a sparse subset of loci and in low order (e.g. pair-wise) interactions. We find that focusing on interaction between loci that exhibit strong additive effects is an efficient way of identifying most of the epistasis. Our model study defines a theoretical framework for interpretation of experimental data and provides statistical predictions for the structure of genetic interaction expected for moderately complex biological circuits.

## Author Summary

Heritable phenotypic properties are often defined by complex pathways and therefore dependent on multiple polymorphisms affecting different genes. Mapping phenotypic consequences of such genetic variation is central to our understanding of disease susceptibility and is fundamental to understanding evolutionary dynamics. How does the effect of multiple genetic polymorphisms occurring together relate to the effect of same polymorphisms in isolation? It is often assumed that individual effects add without interference, yet interactions between polymorphisms have been observed in numerous contexts. The extent to which interactions shape phenotype distributions depends on the nature of interaction intrinsic to the biological system and on the distribution of polymorphisms in the population. Here we approach the systems aspect of the problem by using quantitative modeling of a moderately complex bio-molecular pathway - invertebrate phototransduction - to provide a statistical characterization of non-additive effects of multiple parameter changes. We find that interaction is associated with small subsets of polymorphisms and demonstrate that focusing the study on the set of strong additive polymorphisms accounts also for a significant fraction of total interaction: a finding relevant to the genome-wide-association analysis.

## Introduction

Molecular genetics and systems biology have taught us that cells and organisms function as interacting molecular networks [1], [2] Yet, population genetics studies of correlations between phenotypic traits and genotypes find that phenotypic variation is to a surprising degree attributable to alleles acting independently of each other – an effect largely responsible for the heritability of traits in sexually reproducing populations [3], [4]. Understanding how strongly interacting molecular-genetic networks come to behave this way on the population level remains a fundamental open problem. To make progress we need to understand a) the extent of interaction between specific alleles at different loci which defines the so called “ *physiological* epistasis” [5]; and b) the extent to which epistatic sets of alleles appear in natural populations as manifested by the epistatic component of the observed genetic variance – the so called “ *statistical* epistasis” first defined by R.A. Fisher [6],[7]. The latter would, on the theory side, require understanding of the dynamics of natural selection in population in the presence of epistasis and recombination [8], [9]. Here we shall focus on the question of physiological epistasis: given a set of alleles that affect a given trait, what can we say about the probability of finding a certain level of genetic interaction? Direct measurements of physiological epistasis among mutations at multiple loci has become feasible only recently [10], [11] and still present a formidable challenge. Theoretical investigations of the interactions can in this context provide a useful insight into the expected *generic* behavior. Previous work, for example, has used metabolic flux analysis combined with the quantitative genetics approach [4] to investigate (within models of metabolism) the molecular basis of dominance ([12]–[14]).

Present work is based on the idea that alleles at different genetic loci can be represented by discrete values of different parameters of a mathematical model describing the behavior of a molecular network. This enables a systematic exploration of the “genotype” to “phenotype” mapping represented by the model and a quantitative characterization of the strength of epistasis that may be expected for different allele sets. As a representative example we shall consider invertebrate phototransduction [15] which allows us to take advantage of a recently developed quantitative model of this moderately complex system [16]. Invertebrate phototransduction involves a G-protein and phospholipase-C mediated signaling cascade which in response to the absorption of a single photon generates a spike-like “Quantum Bump” depolarization caused by transient 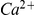 influx into the cell. The magnitude and latency of this response are two examples of quantitative traits associated with this system. The model, which has been demonstrated to capture quantitatively the properties of the system, involves a considerable number of parameters quantifying protein concentrations and the kinetics of molecular processes. To the extent that these numbers are ultimately encoded in DNA sequence that defines the relevant proteins and controls their expression, it is reasonable to associate each model parameter with a genetic locus and we shall simply assume that different “alleles” at that locus correspond to different numerical values of the parameter. It is of course possible that a given parameter is affected by more than one locus, but because this generalization is straightforward we shall assume a simple one to one relationship. For the same reason, we shall not explicitly consider diploidy and dominance and work within a haploid model. Introducing two alleles, i.e., coefficients, at each of 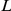 different loci, leads to a representation of the system as one of the 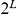 points of a 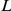-dimensional hypercube. For a moderately large value of the number of loci, the behavior of the system, i.e. any of the quantitative traits and hence its phenotype, can be determined numerically. With this procedure, one can construct the complete genotype to phenotype mapping.

It is useful to set the problem of epistasis into the context of the general problem of understanding parameter dependence of quantitative traits as described by systems biology models. In most cases this dependence is characterized through the sensitivity analysis [17] which examines response to small perturbations about the operating point in the parameter space typically identified via fitting. Recent work [18]–[20] has found that the sensitivity to small perturbations generically possesses “sloppy” modes: local directions in the multidimensional parameter space that have very little effect on the trait in question. The problem considered here is different in two ways. First, as we shall see below, non-trivial epistasis arises when alleles under consideration correspond to very different values of the parameter, so that epistasis involves global dependence on the parameters, rather than the local sensitivity. Second: our definition of epistasis on a hypercube embedded into parameter space aims to extract information relevant to population genetic context (e.g. the case of sexually reproducing population) where parameter space is explored in a combinatorial way through re-assortment of discrete alleles, corresponding to the genetic polymorphisms that exist in the population.

The strength of phenotypic variation induced by an allele emerges as a key parameter that characterizes the genetic landscape of the trait [13]. Consider a locus with an allele pair corresponding to parameter values with ratio 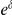. Interaction between several such loci can, in the limit of small 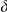, be very simply understood mathematically in terms of the Taylor series. This limit in the leading order of course also reproduces the sensitivity analysis ([1618]). In this limit, the additive part of the gene interaction goes as the first power of 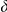, whereas the terms representing the epistatic interaction between 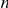 loci scales as 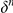. In this sense, non-additive parts simply reflect the effect of nonlinear character of the relation between genotype and phenotype. At larger 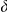 the extent to which the effect of an allele at one locus depends on all other loci, i.e. on the genetic “background”, can be substantial, but varies considerably between loci. We shall provide a quantitative characterization of these epistatic effects. Making connection with the problem of genotype/phenotype mapping commonly encountered in the Genome Wide Association [21]–[24] studies, our analysis suggests that relatively strong additive loci form a good reduced set for investigating epistatic contribution to genetic variance [25].

## Model

### Invertebrate phototransduction system

**Figure 1:**
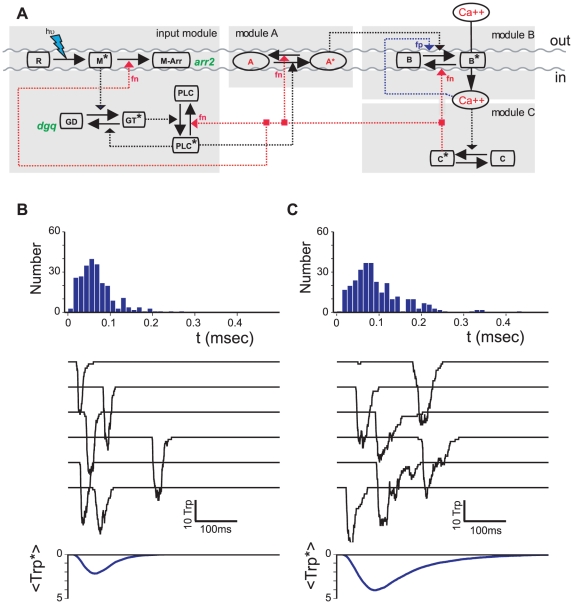
Invertebrate phototransduction: Molecular network and phenotypic variations. Panel (a) provides a schematic summary of molecular mechanisms underlying phototransduction [15], [32]. A photon absorbed by a rhodopsin receptor leads, via 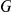 proteins, to the activation of 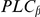, which in turn leads to the production of diacylglycerol (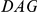), which acts directly or indirectly as the activator 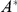 (module A). The activator leads to the opening of 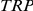 channels (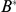) causing a rapid influx of 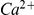 which at moderate concentration reinforces activation thus providing a positive feedback (fp) (module B). At higher concentration 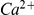, acting via a 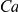
- binding intermediary (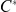), provides the negative feedback (fn) which terminates the Quantum Bump (QB) (module C). Panels (b,c) present samples of computer simulated QBs obtained with a quantitative model [16] of the processes shown in (a). The traces show the number of open 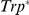 channels as a function of time for 6 different QB. Significant trial to trial variation is observed, in particular in the QB latency time, histogram of which is shown at the top of each panel. Panels (b) and (c) correspond to two different sets of model parameters. This results in quantitative changes in response phenotypes such as QB average duration, latency, amplitude and failure probability (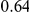 for (b) compared to 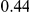 for (c)).

Our theoretical analysis is based on a quantitative model of invertebrate phototransduction [15], [26], [27] developed by the authors and described in Ref. [16]. The absorption of one photon occurs in one of the many (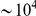) microvilli compartments of a retinal cell and leads through a cascade of reactions to a transient inward electric current, called quantum bump (QB). The signaling cascade can be adequately described by a network consisting of four main modules, represented in Fig. 1a. The input module starts with the activation of rhodopsin by a photon, leading to metarhodopsin, which then catalyses nucleotide exchange on the 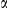-subunit of a heterotrimeric 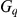 protein. The activated 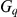 protein activates one phospholipase 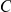 (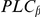) molecule. 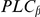 induces hydrolysis of 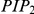 leading to the production of diacyl-glycerol, 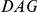, which acts (directly or indirectly) as the activator (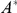), see “module A” in Fig. 1a. The activator 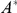 induces the opening of the 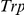 channels, represented as the transition from 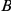 to 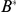 in “module B” (Fig. 1a), which lets 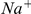 and 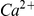 flow into the microvillus. At moderate concentration, the influx of 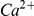 triggers a positive feedback, which opens more 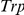 channels, and further increases calcium concentration. At higher intracellular calcium concentration, the negative feedback (module C), due to formation of the molecular species 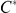 catalyzes the closing of 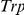 channels, as well as the termination of the activation of several key elements, leading to the return of the cell to its quiescent state. One important aspect of the response of the system to the absorption of one photon is its strongly stochastic character. This property is a consequence of the very small number of some of the key molecular species participating in the signaling cascade. The response of the system should thus be characterized by its statistical properties. The simulations of the model have been shown to reproduce quantitatively the single photon response phenotype of the wild-type receptor, as well as the proper dependence on external calcium concentration. As demonstrated in Ref. [16] the model also captures the behavior the known mutants, such as those with impaired metarhodopsin deactivation, or with strongly reduced expression of 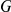-protein. In the model, such mutants are represented by suitable variations of one of the parameters [16].

Following the success of the model in representing mutant phenotypes we assume that systematic study of phenotypes corresponding to sets of discrete parameter values can provide insight into the expected extent of physiological epistasis. Let 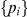 with 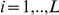 denote a set of 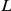 parameters characterizing the model system and 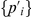 be an alternative set. We can think of the two pre-assigned parameter values 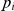 and 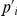 as the two “alleles” at the locus “
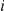
” and construct 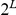 parameter sets consisting of all possible combinations of these alleles: each set corresponding to a different genotype. Our phototransduction model has 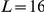 parameters, giving rise to 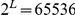 possible “genotypes”. As expected, most of these different “genotypes”, i.e. parameter combinations, correspond to different phenotypic responses, as illustrated in Fig. 1b,c. The probability to elicit one QB is smaller for the genotype shown in panel 1b, than for the genotype in panel 1c. Similarly, the latency of photoreceptor response is significantly shorter for the genotype (1b) than for (1c). The individual QBs are narrower for genotype (1b), and have a slightly larger amplitude than the QBs generated by genotype (1c). As a result, the average over many QBs leads to a smaller amplitude and a shorter duration for genotype (1b), compared to (1c). We note in this respect that the method we use here to study the system is a generalization of the approach based on random variations of the parameters, introduced in [19].

How different the phenotypes are expected to be depends quantitatively on the difference between the parameter values that define the alleles at each locus. This difference is characterized here by a positive parameter 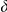 in the following manner: starting with a parameter set 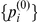 we generate “alleles” 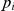 and 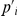 via 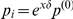 where 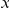 is a Gaussian random number, with zero mean and unit variance (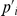 being defined in the same way but with a different random 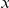). As a result, the typical difference 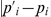 scales as 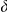 when 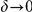. The value of 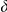 will play an important role in our discussion of epistasis.

The performance of the photoreceptor cells can be used to define some simple traits. For example, reliability of the response to one photon, defined as the probability that one photon elicits a QB is a possible characterization of the response of the system. This probability, a number between zero and one, defines a “quantitative trait” 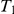. Alternatively, one may consider the averaged response of the photoreceptor over many incident photons, which corresponds to the cumulated response of the many microvilli composing the photoreceptor cell. Typical signals, shown as the lowest traces in Fig. 1b and c, can be characterized by their amplitude 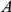 and duration 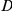. The amplitude in our model corresponds to a number of open 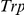 channels; in practice, it corresponds to the recorded peak current obtained after a flash of light is sent to the cell; it is typically of the order of 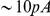. The duration of the pulse is typically of the order of 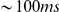. The two associated traits, 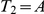 and 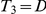 are thus dimensionful quantities, setting the scales respectively for the current and for pulse duration. In addition to these simple traits, one may define composite traits, involving a combination of several statistical properties of the response of a cell to one photon, based on tradeoffs that may be advantageous for the system. For example, it may be intrinsically interesting for the system to generate a response both with high amplitude and a short duration. The corresponding trait may be defined as the product of two sigmoid functions via 
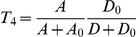
 defined relative to parameters 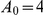 and 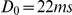. The four traits introduced above are just a few out of many possibilities. The general picture that emerges from the study presented here was found to be essentially independent of the particular trait considered.

### Mathematical model of the invertebrate photo-transduction cascade

Following Pumir et al [16] we describe the phototransduction cascade by the following set of chemical kinetic equations. In the following, starred variables refer to activated molecular species. Once rhodopsin has been activated by light absorption (forming metarhodopsin), it deactivates according to:
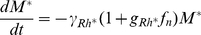
(1)


Active metarhodopsin in turn activates G-proteins according to:
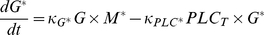
(2)


The activated G-protein deactivates by reacting with PLC and becomes inactivated, before returning to its resting state, 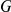:
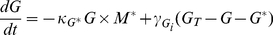
(3)


Activation of 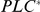 is governed by:
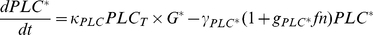
(4)


and leads to the production of activator molecules 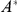:
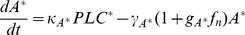
(5)


which in turn opens 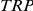 channel (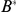 denoting the number of open 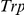 channels).
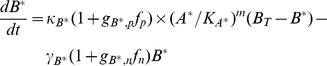
(6)


As a result of the opening of 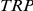 channels, Calcium enters the cell, according to:
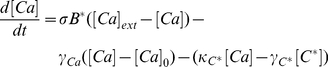
(7)


where 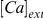 and 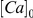 refer respectively to the external and to the intracellular equilibrium 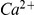 concentrations. Finally, intracellular 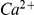 activates an inhibitor, 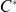, according to:
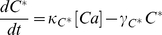
(8)


Opening and closure of channels, as well as several steps in the cascade are modulated by the positive, 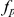 and negative 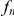 feedbacks, simply parametrized in terms of a Hill function:
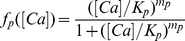
(9)

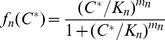
(10)


The kinetic coefficients of the model have been determined by imposing that the statistical properties of the response of the receptor to a single photon are correctly predicted by the model. The equations that define the model involve a number of coefficients, which quantify the activation and deactivation rates, as well as the various feedbacks that play a crucial role in the process. Reference values of all parameters are as given in Table 1 in Text S1, see also [16].

### Numerical method

The stochastic version of kinetic equations was simulated via Gillespie algorithm as described in [28], and used in [16] with statistical averages taken over about 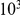 response realizations for each set of parameters. Averages was taken over 20 sets of randomly generated alleles at 16 loci of the system for five values of 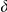 (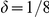, 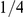, 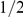, 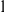, 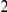). At small values of 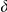, where alleles correspond to only small changes of corresponding parameter pairs, and the difference in the trait can become comparable to the intrinsic variance of the response. For this reason, we used more realizations to compute the traits at small values of 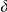: specifically the number of response trials was 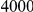 for 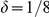 and 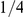; 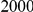 for for 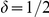 and 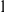; 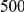 at 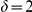). The simulation was implemented using Fortran and ran on a PC. The time necessary to compute all trait functions, for all genotypes was 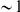 day. Once determined, the trait function on the hypercube was decomposed according to Eq.1, using a Fast Fourier Transform, with the result defining the “spectra” 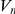 and 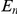.

### Quantitative representation of the genetic interaction

The advantage of our model system is that it allows us to determine numerically the full mapping between the genotype and phenotype. To this end, we begin by representing a vertex on a 16-dimensional hypercube by using for each allele a variable 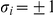. Any quantity characterizing the phenotype of the system (such as the traits introduced above) associated with the genotype 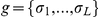 can in full generality be represented by [29]:
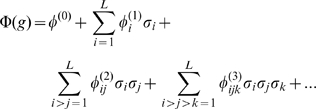
(11)


Here, the first term is the constant component of 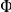 which does not depend on the monitored loci. The second term corresponds to a sum over independent contributions of the 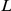 loci. Subsequent terms parameterize the contribution of all pairs, triplets and higher multiplets of loci. For example, the second sum runs over all pairs of loci and for each pair, 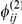 specifies the dependence of fitness on locus “i” when the “
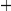
” allele at locus 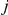 is replaced by a “
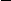
” allele, averaging over all other loci with uniform probabilities. These higher order terms quantify the deviations from a strictly additive contribution of different loci to the traits, corresponding to *physiological epistasis*. The representation (11) is the most compact parameterization of arbitrary functions that can be defined over the 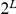 possible genotypes. All such functions are uniquely defined by the set of 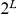 parameters 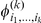 (with 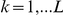). Equation (11) can also be interpreted as the Fourier transform of the phenotype function defined on a hypercube. This framework can be generalized to more than two alleles per locus, and to the diploid context. Note that in contrast to [30] and consistent with idea of physiological epistasis, the parameterization (11) is explicitly independent of genotypic distribution (allele frequencies) in the population.

The representation of the phenotype function via Eq.11 provides (by analogy with the Fourier transform) a simple measure of the distribution of epistatic interaction among different orders: we define an “epistatic power spectrum” as the average variance of all 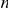-tuple interactions:
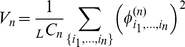
(12)


where 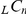 stands for the 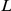-choose-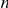 binomial coefficient - the number of 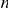-tuplets in the sum. Quantitative genetics [3], [4], [31] representation of epistasis often assumes, tacitly or explicitly, that epistatic contribution to the phenotype is such a complex function of the genotype that it looks essentially random. In that case 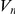 is constant and the *total* variance contributed by 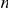-tuple interactions, 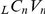, increases rapidly with 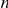 along with the number of terms contributing to Eq. (12). More generally because the number of possible 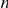-tuplets increases rapidly with 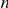, multi-loci interactions could dominate the total phenotypic variance even if on average 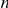-tuple interaction terms, 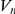, are small [5]. Below we will show that this does not happen in the realistic system that we consider.

If one focuses on a particular locus, say locus 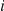, it makes an additive contribution 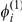 to the trait with the rest of its effect dependent on the genetic background, i.e. on alleles at all other loci. Correspondingly we define the additive variance 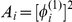 associated with locus 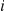 and the epistatic variance
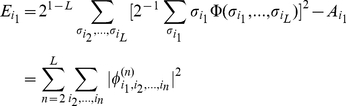
(13)


These definitions will allow us to demonstrate that, independent of particular alleles, some loci are more likely to exhibit background dependence or epistasis than the other.

## Results

To identify the extent of “physiological epistasis” in the phototransduction model we computed the properties of the single photon response 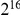 “genotypes” defined by all possible combinations of the pairs of parameter “alleles” 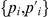 defined relative to a reference set 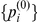 by random rescaling (e.g. 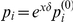, 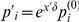)) with the typical magnitude controlled by 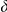 as described above. This defined a genotype to phenotype map for a particular allele set. The procedure was then repeated 20 times for random allele sets and the results given below, unless stated otherwise, are statistical averages over this ensemble.

**Figure 2:**
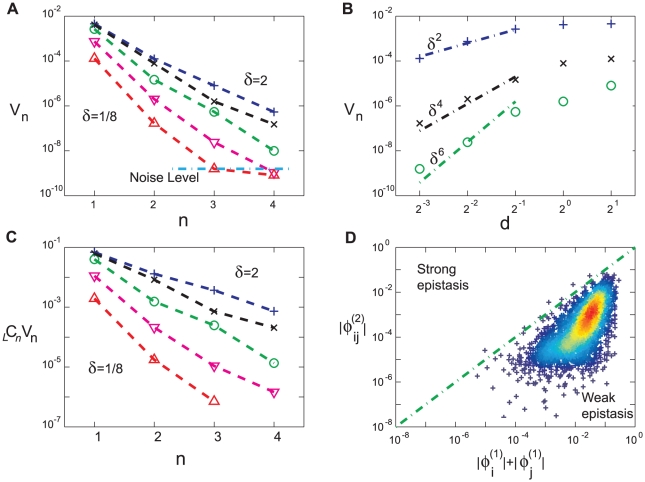
Quantitative characterization of the multi-locus interaction. The “epistatic power spectrum” defined by Eq. 12,13 characterizes the strength of the 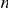-loci interactions of the trait. Panel (a) shows the variance 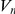 for several values of 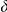. As 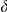 and hence the difference between the parameters, becomes smaller, the exponential decay of 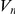 with increasing 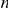 becomes faster. Determination of 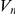 for large 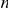 and small 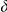 is limited by the noise, caused by finite sampling errors when computing the trait. Panel (b) shows the dependence of 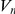 (for 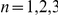) on 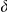. For small 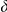 the variance 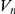 scales as 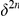. Panel (c) shows the total variance in 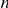-locus interaction. Large number of contributing multiplets partially compensate for the smallness of the average contribution (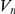) making epistasis for larger 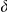 comparable with the additive variance (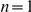); nevertheless total epistatic variance decreases with the order of interaction even for the largest 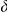 considered. Panel (d) shows a scatter plot of the 2-epistasis term 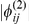 vs. the additive part of the interaction 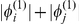, see text, for all pairs 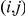 of loci, and for 20 different choices of the alleles, corresponding to 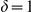. The color reflects the local density of points; it is high (low) in the red (blue) region.

The mean variation of the 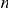-loci epistatic term 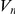 is shown in Fig. 2a for the trait 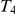. For each value of 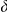, 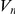 decays approximately exponentially with 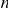. The decay rate however decreases with increasing 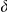. In the very small 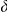 limit, as explained above, the term 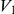 is expected to be proportional to 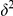, and multiplied by the magnitude of the tangent map considered in the sensitivity analysis [19]. The higher order terms (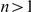) decrease exponentially. Accurate determination of the small 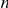-loci epistatic contribution, for high 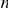, is limited by the noise due to stochastic nature of the response and the finite size of the sample. The noise level indicated in Fig. 2a was estimated by comparing the averages using two different values of the number of QBs. Rapid decay of 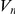 with 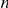 when 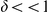 implies that the additive term in the representation given by Eq.1 is the dominant one, so in this limit epistasis is very weak.

Fig. 2b quantifies the effect more precisely, by showing the same set of data, plotted as a function of 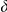 for different values of 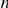. The main conclusion is that for small values of 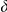, 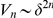. This behavior has a simple interpretation. The traits studied here are functions of the model parameters. Assuming that these functions can be expanded in Taylor series, and noting that in the limit 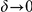, the difference between the parameter alleles 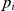, 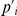 at each locus scales as 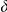. A straightforward expansion of the trait function then shows that the term 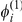 can be expressed as a derivative of the trait function with respect to 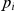 (evaluated at 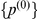) times 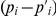: hence 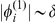 and 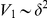. Similarly, the term 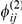 is proportional to the second derivative of the trait in the directions 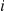 and 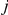, multiplied by 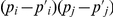. This implies that 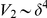 and more generally 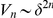 thus explaining our numerical findings in the small 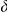 regime. We conclude that quite generally, for alleles corresponding to small perturbations of the system epistatic interaction between loci is much smaller than the additive effect of the loci.

The total variance due to 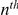 order epistasis, as measured by 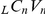 is shown in Fig. 2c. Epistasis is weak for 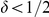. More surprisingly we observe that total variance due to 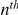 order epistasis decreases with 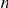, even for the largest value of 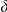 studied (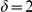), although less and less rapidly as 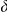 increases. (Note that 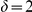 corresponds to about 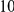-fold difference between parameters corresponding to any two alleles.) Additivity remains quite strong: 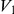 accounts for 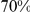 of the total variation for 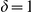, and about 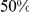 for 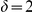, yet even for 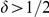 a significant part of the variation comes from the interactions between loci.

**Figure 3:**
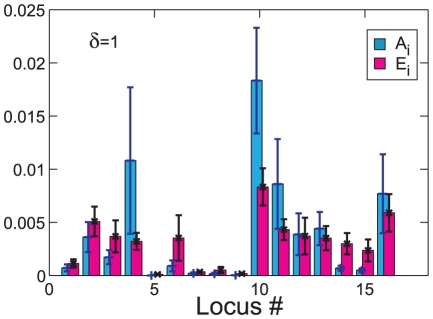
Variation between loci. The additive 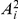, and the background dependent (i.e. epistatic) contributions, 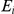 as defined in the text, are presented locus by locus, obtained by averaging over 20 sets of random alleles (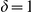). The relative strength of epistatic and additive components varies widely among loci. While a few loci make a stronger epistatic than additive contribution one notes that loci with the strongest additive components also have the strongest epistatic components. Note that sample to sample fluctuations of 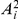 and 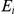 are very large, resulting in the large error bars shown in the figure.

We find extensive variability in the strength of epistasis even between terms of the same order (i.e. between different 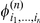) so that only a small subset of possible 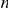-tuplets contribute with the strength 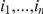 much greater than the average, 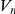. This effect is illustrated in Fig. 2d, which for a given pair of loci (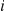, 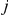) compares the strength of the additive component 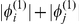 with the strength of the interaction between the same two loci: 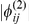. Fig. 2d presents a scatter plot for all pairs of loci and 20 sets of alleles (with 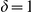). The dashed line corresponds to 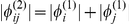 so that points lying above this line correspond to strong interaction between the loci. Color codes for the density of points: red correspond to the highest density and blue for the lowest density of points. The red region gives a good idea of the average of the epistatic contribution, conditioned on the additive part (see also Fig. 1 in Text S1). In the vast majority of cases, pair epistasis is small compared to the additive contribution. However, the epistatic component is comparable to the additive term for a significant fraction of all configurations: 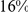 of all possible pairs of loci correspond to an epistatic contribution in excess of 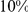 of the additive part. A similar picture emerges in considering epistasis among triplets of loci (see Fig. 2, Fig. 3 in Text S1).

**Figure 4:**
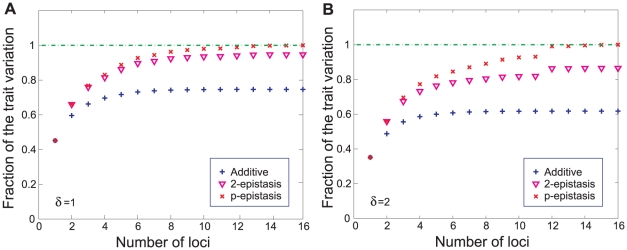
Inference of the strength of epistasis from a restricted subset of loci. Blue 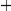 signs show the cumulative fraction of the trait variation due to the additive component; purple inverted triangles show the cumulant of the additive component plus the 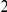-loci epistasis (involving only the strongest loci); and red crosses show the cumulant of the total trait variation. Panel (a) 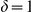 and panel (b) 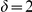. The cumulative sums are computed with the 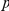 loci that have the largest additive contribution to the trait. The relative contributions are all divided by the total variation observed for the complete set of 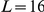 loci.

The variability of the epistasis is not just the matter of specific alleles. It reflects the properties of the network in the sense of some pairs of loci are much more likely to interact than others (see Fig. 4 in Text S1) – a property that can be quantified by averaging over the large number of possible allele sets. Alternatively we can compare the background independent contribution 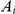 of a given locus to the background dependent, epistatic, component 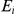. As we see in Fig. 3, some of the loci contribute much more than others, although this variation is more pronounced for the additive component 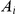. The large variation in the contribution of different loci seen in Fig. 3 is consistent with the large variation of parameter sensitivities noted before for generic systems biology models [18], [19] and for this system in particular [16]. We reiterate however, that the connection between local sensitivity and the global analysis presented here is far from obvious.

### Implications for genome wide association studies

The genome wide association studies (GWAS) use linear regression between quantitative traits and genetic polymorphisms to identify loci that contribute additively to the phenotype [23], [24]. It is not uncommon that thus identified additive loci account only for a small fraction of genetic variance. The remainder of the variance could be associated with small additive contributions of a very large number of loci, with rare strong alleles and with epistasis. Brute force detection of epistatic effects by including in the regression all pairs of loci runs into the multiple hypothesis testing problem and is not feasible. It was however found that loci identified by their additive effects, also exhibit substantial pairwise epistasis [25]. Yet, it is not known if focusing on the additive loci could be expected to capture a significant fraction of total epistatic variance. This question however can be investigated in context of the present model. To that end we shall rank loci by their additive contribution to the trait and consider a subset of 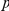 top loci. We then define epistatic variance associated with this subset as a sum of squares of the 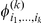 terms for all combinations of loci within the subset. Fig. 4a,b present the cumulant additive, pairwise epistatic and total epistatic variance for 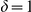 and 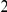 (trait 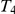 averaged over 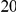 allele sets). We observe that for the system under consideration the additive contribution is dominated by the 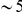 loci with the strongest additive components. Focusing on these loci for 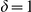 and 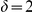 one recovers respectively 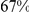 and 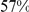 total epistatic variance. Note that consistent with the results shown in Fig. 2, pairwise epistasis provides the main contribution to the total epistasis. It is quite remarkable that the inclusion of loci with weaker additive effects adds less epistasis: this is almost always true, although in Fig. 4b we note that locus #12 with low additive variance contributes much more epistasis than comparable loci. This general behavior can be understood by noting that provided that epistatic power spectrum decays rapidly with the order, the additive contribution will generically be comparable to the total. Suppression of the additive component would require some accidental symmetry to make the effect of the allele average out to zero over all possible genetic contexts.

## Discussion

We have used a quantitative model of signal transduction to develop a computational approach to study the nature of interaction between multiple parameters representing key components and reactions of the pathway on the molecular level. Our approach relies on the quite general assumption that these bio-chemical parameters are defined by the structure and expression level of proteins and hence are encoded genetically. Thus we assume that a genetic polymorphism corresponds to a parametric polymorphism. Our interpretation of parameters as loci and of discrete values that they take as alleles is an abstraction in the sense that we do not connect model parameters with specific genetic sequences. Instead we have formulated and focused on questions that are independent of the specific connection between parameters and sequences.

As expected we find that epistasis is weak for parameter alleles quantitatively similar to each other. Less obviously, we find that the strength of epistasis decays exponentially with the order of interaction even when alleles correspond to large changes in system parameters. Epistasis involves only a small fraction of possible subsets of loci and is dominated by pairwise interactions. Yet the number of interactions is sufficiently large that the total contribution can be substantial even if individual terms are small. Our findings with regard to weak alleles are clearly general and can be rigorously ascertained via their connection with the Taylor expansion. The generality of the conclusions for strong alleles (i.e. 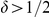) is supported by the fact that they are independent of the quantitative trait considered. We expect that our observations concerning the decay of epistatic spectrum will hold also for generic systems with much larger number of parameters or loci: study of systems with larger 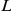 would be an interesting subject for future research.

Our results are consistent with the expectation based on sensitivity analysis and the observed structure of the “tangent map” corresponding to very small variations of the parameters [20]. As already noted, the correspondence is direct in the limit of weak alleles (i.e. small 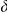). The eigenvectors with the largest eigenvalues of the tangent map define a few directions in the (multidimensional) phenotypic space, which are most strongly affected by variations of the coefficients: any small variation of the parameter thus leads to a deformation of the solution in these directions. For stronger (but not too strong) alleles, nonlinear effects giving rise to epistasis are weak and can be understood mathematically using perturbation theory, as explicitly done in the Supplementary Text S1. Generally within perturbation theory one expects that epistasis will affect mostly pairs of parameters that contribute to two different eigenvectors with large eigenvalues. In this respect, the identified structure of the “genotype to phenotype” mapping provides a natural explanation of the fact that the parameters that contribute most at the additive level are also those that contribute most to the epistasis. For larger 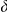 when the potentially complex (non-linear) parameter dependence comes into play the correspondence between the local and global structure cannot be assumed. (Note that Fig. 4 correspond to large - 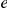-fold (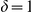) and nearly 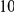-fold (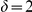) variations of the parameters.) Yet at present rigorous global characterization of parameter dependence in the general class of models considered here remains a non-trivial mathematical challenge. Of many possible ways to extend the linear sensitivity analysis, our choice of considering discrete sets of alleles was inspired by the case of genetic and phenotypic variation in sexually reproducing population.

Epistatic power spectrum, defined naturally through the Fourier transform analogy of our parameterization of the genotype to phenotype map (11), provides a very general way of characterizing the extent of epistasis. It would be interesting to develop mathematical ideas that could provide a classification of dynamical systems, such as bio-chemical networks modeled here, and generate bounds on the strength of “parametric entanglement” as characterized by the epistatic spectra introduced here.

Epistatic interactions involving more than two loci are a manifestation of nonlinearities occurring in a genotypic space involving combinations of alleles with large phenotypic effects. Such nonlinearities are clearly observed experimentally in the binding affinity of transcription factors; see [11]. More experimental studies providing quantitative measurements of phenotypes for defined combinations of alleles at multiple loci are needed in order to bridge our understanding of protein interaction in the system biology context and genetic interactions which play a role in heritability of phenotypes in sexually reproducing populations.

Finally, we stress the conclusion that a significant fraction of epistasis can be found by focusing on the loci identified by their additive contribution: this notion has immediate practical implications in the context of GWA studies.

## Supporting Information

Text S1 Supplementary materials.
(1.76 MB PDF)
