# Supplementary material for: Epistasis in a Model of Molecular Signal Transduction
Source: PLoS Comput Biol. 2011 May 12;7(5):e1001134. doi: 10.1371/journal.pcbi.1001134 (PMC3093352; doi:10.1371/journal.pcbi.1001134)
Supplement: Text S1 — Supplementary materials. (1.76 MB PDF) [file pcbi.1001134.s001.pdf]

Supporting information for
"Epistasis in a model of molecular signal transduction"

Alain Pumir and Boris I Shraiman

1 List of 16 model parameters set used in the study (see Methods)

Values given are the reference set $\{p_i^{(0)}\}$, based on [1].

$\gamma_{B^*} = 6.0 \times 10^{-2}$	Deactivation rate of TRP/TRPL channels (B^*) (ms^{-1})
$\gamma_{A^*} = 2.1 \times 10^{-2}$	Deactivation rate of activator (A^*) (ms^{-1})
$\gamma_{Ca} = 33$	Rate with which Ca is pumped out (ms^{-1})
$\gamma_{PLC^*} = 4.8 \times 10^{-2}$	Deactivation rate of PLC^* (ms^{-1})
$\gamma_{G^*} = 3.5 \times 10^{-2}$	Deactivation rate of G^* (ms^{-1})
$\gamma_{C^*} = 5.5 \times 10^{-3}$	Deactivation rate of C^* (ms^{-1})
$g_{B^*,p} = 41.5$	Strength of the positive Ca feedback on Trp
$g_{B^*,n} = 5.4$	Strength of the negative C^* feedback on Trp
$g_{PLC^*} = 11.1$	Strength of the negative C^* feedback on PLC^*
$g_{A^*} = 37.8$	Strength of the negative C^* feedback on A^*
K_{A^*}<br>$\kappa_{B^*}/K_{A^*}^3 = 1.310^{-6}$	Characteristic scale of A^* for activation of Trp channels<br>rate: $\kappa_{B^*}/K_{A^*}^3$ (ms^{-1})
$\kappa_{C^*} = 1.0 \times 10^{-2}$	Rate of C^* activation (ms^{-1})<br>Expected to be proportional to Ca concentration
$\kappa_{PLC^*} = 3.9 \times 10^{-3}$	Rate of PLC^* activation (ms^{-1})
$\kappa_{A^*} = 7.4 \times 10^{-2}$	Rate of activator A^* production (ms^{-1})<br>Expected to be proportional to PIP_2 concentration
$\kappa_{G^*} = 4.7 \times 10^{-3}$	Rate of G^* activation (ms^{-1})
$\sigma = .17$	Influx of Calcium ions per TRP/TRPL channel (ms^{-1})
$K_n = 19.5$	K_D for negative Calcium feedback (mM)

2 Epistasis for moderate values of δ: why loci with highest epistasis tend to be those with highest additive effect.

The analysis of Ref. (1) identifies, in the limit of small perturbation of model parameters ($\delta \to 0$ in the notation of our main text) the dominant eigen-directions in the parameter space, which maximize (linear) sensitivity. Our analysis of epistasis focuses on non-linear effects. In the regime of small parameter perturbations, one can identify epistasis with nonlinear (i.e. non-additive) corrections to the response, which appear formally at next order in the systematic weakly nonlinear expansion. Our notation here follows the notation of Ref. [2]). Consider the mapping M that associates to a change in the (scaled) parameters $\delta\eta$ the induced change in the (multidimensional) solution space, δQ.

$$\delta Q = M \cdot \delta\eta \tag{1}$$

The number of model parameters is denoted by s. Effectively, the solution space is discretized, so as to provide a finite dimensional mapping from the s-dimensional parameter space to the discretized solution space, say of dimension N. Without loss of generality, we assume $N > s$. The operator M then can be thought of as a $N \times s$ matrix (with s columns and N rows). Using standard Singular Value Decomposition techniques, the matrix M is decomposed in the form:

$$M = UDV^{\dagger} \tag{2}$$

where V is an orthogonal s-by-s matrix ($V.V^{\dagger} = V^{\dagger}.V = Id_s$), $D = diag(\sigma_1, \sigma_2, ...\sigma_s)$ is a diagonal matrix, and U is a s-by-N matrix. The interpretation of this decomposition is that V defines the eigen-directions in the parameter space, given by the images by the matrix V of the basis vectors $\hat{\mathbf{e}}_1$,$\hat{\mathbf{e}}_2$, ..., $\hat{\mathbf{e}}_s$. Each of these directions is associated with one of the eigenvalues σ_i. The nontrivial observation of [2] is that the spectrum of eigenvalues σ_i decays very rapidly, so that assuming $\sigma_1 \geq \sigma_2 \geq ... \geq \sigma_s$, only a small number of directions in the space of solution are actually affected by variations of the parameters.

Based on this analysis, it is easy to see that the eigenvectors in the parameter space specify the parameters with the largest influence on trait variations. The parameters that have the largest components on the eigenvectors associated with the large eigenvalues are the one that also have the largest "additive effect" on the traits we are considering, at least in the small δ limit.

This has been stressed by Rand, who has proposed several tools to represent which parameters contribute to which eigen-directions [3].

All the considerations so far addressed the linear response of the trait in the limit of very small parameter changes. Understanding what happens when the value of δ is small, but not infinitesimal can be easily done by pushing to the next order in a Taylor series expansion of the trait, or more generally, of the solution. Given one particular trait, the linear approximation leads to the conclusion that the trait/solution variation results from a mixture of the various eigenmodes. On general grounds, one expects that the eigen-directions with the largest eigenvalues are the one that will contribute most to the trait variation: an arbitrary change of the model parameters results in an "ellipsoid", with the largest axes corresponding to the largest eigenvalues [2].

The largest eigen-directions affecting the trait are thus most likely to result in a nonlinear effect. To quantify this effect, it is useful to make a change of variable, and to take the eigen-directions in parameter space as new coordinates. The straightforward procedure thus consists in introducing a new coordinate system, (λ_1 ,λ_2 , ..., λ_s), defined locally by:

$$\delta\lambda_i = \sum_{j=1}^{s} V_{ij} \cdot \delta\eta_j \tag{3}$$

where V is the matrix introduced in Eq.2. In this coordinate system, at linear order, a change of the parameters, $(d\eta_1, d\eta_2, ... d\eta_s)$, thus of the values of $d\lambda_i$ results in a change of a trait T:

$$\delta T = \sum_{j=1}^{s} \frac{\partial T}{\partial \lambda_i} \cdot \delta \lambda_i \tag{4}$$

where $\frac{\partial T}{\partial \lambda_i} \propto \sigma_i$. The change in the directions with the largest values of σ are thus associated with the strongest deviations from the linear approximation. This is particularly true for the two largest values of σ_i, σ_1 and σ_2. Including the corrections to the linear order in the variation of the trait due to the two first (strongest) directions leads to:

$$\delta T \approx \sum_{j=1}^{s} \frac{\partial T}{\partial \lambda_i} . \delta \lambda_i + \frac{1}{2} \frac{\partial^2 T}{\partial \lambda_1^2} \delta \lambda_1^2 + \frac{1}{2} \frac{\partial^2 T}{\partial \lambda_2^2} \delta \lambda_1^2 + \frac{\partial^2 T}{\partial \lambda_1 \partial \lambda_2} \delta \lambda_1 \delta \lambda_2 \tag{5}$$

It is straightforward to add more directions than the first two in the equation above. For the sake of the present discussion, it is sufficient to keep only the two first coordinates, λ_1 and λ_2.

In general, the knowledge of the linear term only is not sufficient to make any statement about the size of the higher order derivative terms. However, based on plausible approximations often used in physical systems, it is reasonable to expect that the second derivatives are of order

$$\frac{\partial^2 T}{\partial \lambda_i \partial \lambda_j} \sim \sigma_i \sigma_j \tag{6}$$

Based on this assumption, one would expect that epistasis is strongest either for different parameters that have a strong contribution to the same eigen-direction, or between two parameters that have a strong contribution to two eigen-directions with large eigenvalues, such as directions 1 and 2. This picture provides a very plausible explanation of the observation summarized by Figures 3 and 4, namely that the coefficients with the largest linear contributions, which appear in the largest eigenvectors of the mapping defined by Eq.1 also contribute most to epistasis.

This plausible picture thus provides a heuristic explanation of several of our observations. Still, we wish to point out that:
(i) Eq.6 has been postulated on heuristic grounds; epistasis could be much stronger or weaker depending on the precise values of the higher order derivative terms.
(ii) Our work has been carried out for very strong values of the coefficients variation, so there is no guarantee that the simple Taylor series considerations apply to the case $\delta = 1$ or $\delta = 2$, as suggested by Fig.3 and 4.

References

[1] Pumir A, Graves J, Ranganathan R, Shraiman BI (2008) Systems analysis of the single photon repsonse in invertebrate photoreceptors. Proceedings of the National Academy of Sciences of the United States of America 105: 10454-10359.

[2] Rand D A, Shulgin B V, Salazar J D, Millar A J (2006) Uncovering the design principle of circadian clocks: mathematical analysis of flexibility and evolulionary goals. J Theor Biol 238: 616-638

[3] Rand D A (2008) Mapping global sensitivity of cellular network dynamics: sensitivity heat maps and a global summation law. J R Soc Interface Suppl 5: S59-S69

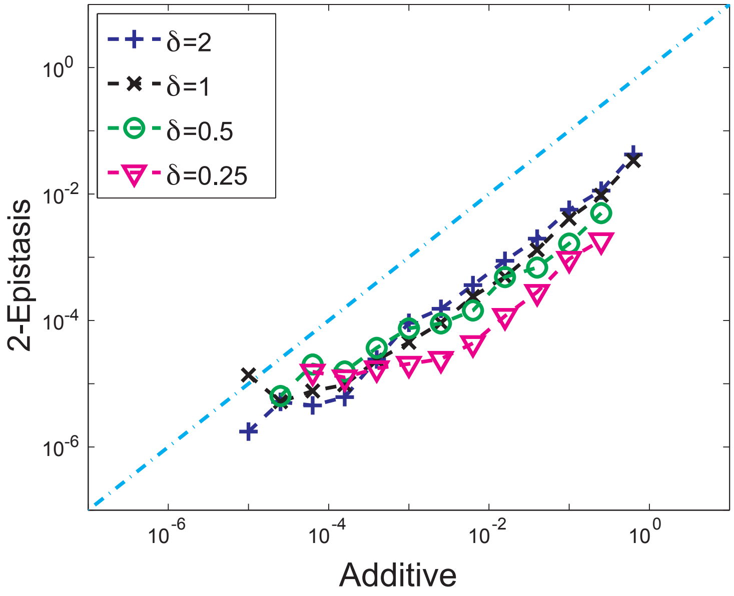

Figure 1: **Figure S1** The mean value of $|\phi_{ij}^{(2)}|$ conditioned on $|\phi_i^{(1)}| + |\phi_j^{(1)}|$ for different values of δ. The relative contribution of the 2-loci interaction increases when δ increases.

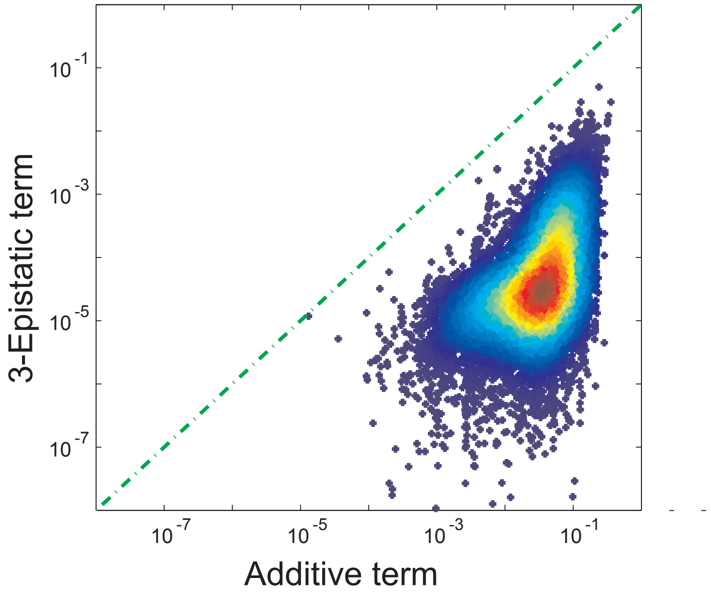

Figure 2: **Figure S2** Scatter plot of the 3-epistasis term $|\phi_{ijk}^{(3)}|$ vs. the additive contribution $|\phi_i^{(1)}|+|\phi_j^{(1)}|+|\phi_k^{(1)}|$ for all triples $(i,\, j,\, k\ )$ of loci, and for 20 different choices of the alleles, corresponding to $\delta = 1$. The color coding reflects the local density of points; it is largest (smallest) in the red (blue) region.

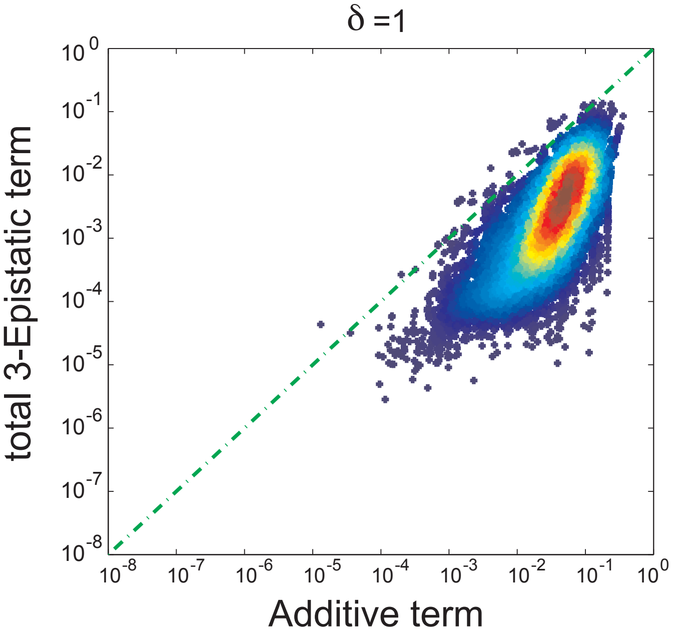

Figure 3: **Figure S3**: Scatter plot of the total epistatic interaction $|\phi^{(3)}_{ijk}| + |\phi^{(2)}_{ij}| + |\phi^{(2)}_{jk}| + |\phi^{(2)}_{kj}|$ vs. the additive contribution $|\phi^{(1)}_{i}| + |\phi^{(1)}_{j}| + |\phi^{(1)}_{k}|$, for all triples $(i,\, j,\, k)$ of loci, and for 20 different choices of the alleles, corresponding to $\delta = 1$. The color coding reflects the local density of points; it is largest (smallest) in the red (blue) region.

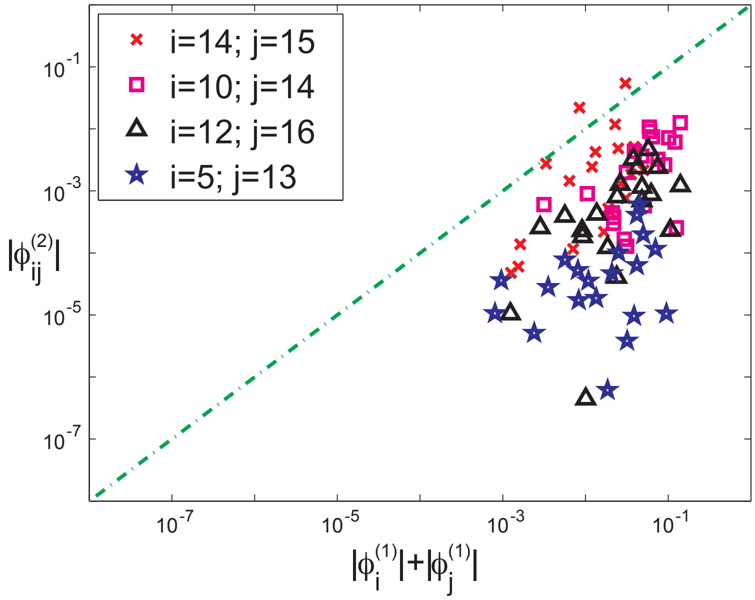

Figure 4: **Figure S4** Scatter plot of $|\phi_i^{(1)}| + |\phi_j^{(1)}|$ vs. $|\phi_{ij}^{(2)}|$, for one pair of loci with a strong (on average) epistatic interaction ($i = 14$, $j = 15$), for two pairs with moderate epistatic interaction ($i = 10$, $j = 14$) and ($i = 12$, $j = 16$), and for one pair with a very weak epistatic interaction ($i = 5$, $j = 13$) . The very large scatter illustrates the wide variability between pairs of loci and different allele sets.
